# An automated instrument for intrauterine insemination sperm preparation

**DOI:** 10.1038/s41598-020-78390-3

**Published:** 2020-12-07

**Authors:** Alex Jafek, Haidong Feng, Hayden Brady, Kevin Petersen, Marzieh Chaharlang, Kenneth Aston, Bruce Gale, Timothy Jenkins, Raheel Samuel

**Affiliations:** 1 Department of Mechanical Engineering, University of Utah, Salt Lake City, UT USA; 2 Nanonc Inc., Salt Lake City, UT USA; 3 Department of Surgery–Urology, University of Utah Andrology and IVF Laboratories, Salt Lake City, UT USA; 4 Brigham Young University, Provo, UT USA

**Keywords:** Biomedical engineering, Mechanical engineering, Lab-on-a-chip

## Abstract

Sperm preparation is critical to achieving a successful intrauterine insemination and requires the processing of a semen sample to remove white blood cells, wash away seminal plasma, and reduce sample volume. We present an automated instrument capable of performing a sperm preparation starting with a diluted semen sample. We compare our device against a density gradient centrifugation by processing 0.5 mL portions of patient samples through each treatment. In 5 min of operating time, the instrument recovers an average of 86% of all sperm and 82% of progressively motile sperm from the original sample while removing white blood cells, replacing the seminal plasma, and reducing the volume of the sample to the clinically required level. In 25 min of operating time, density gradient centrifugation recovers an average of 33% of all sperm and 41% of progressively motile sperm. The automated instrument could improve access to IUI as a treatment option by allowing satellite doctor’s offices to offer intrauterine insemination as an option for patients without the clinical support required by existing methods.

## Introduction

It is estimated that 15% of couples struggle with infertility^1^ and that 1.6% of all births in the US employ some form of assisted reproductive technology^2^. Of assisted reproductive technology methods, intrauterine insemination (IUI) is the least expensive and least intrusive^3,4^. Crucial to every IUI procedure is the required selection and preparation of sperm cells from semen. The first step of all sperm preparation methods is liquefaction, or liquification, in which the highly viscous seminal plasma becomes more liquid^5^. After this step, the preparation process must:

1. Separate the sperm cells from contaminating debris, especially white blood cells (WBCs).
2. Remove the majority of the prostaglandin-containing seminal plasma.
3. Reduce the sample volume to less than 1 mL.

As we compare sperm preparation techniques for IUI, we seek a protocol that recovers a high percentage of motile sperm cells in a short amount of time. The optimal technique would be fast, inexpensive, easy to implement in a clinical setting, and expose sperm to minimal forces.

The clinical gold standard for sperm preparation is density gradient centrifugation (DGC). DGC employs a column of density-stacked fluids to select sperm based on their relatively high density. The main shortcomings of a DGC-based clinical protocol are that it requires 25 min of centrifugation, typically recovers only 40–60% of viable cells, exposes sperm to centrifugal forces that increase oxidative stress and DNA damage^6,7^, requires multiple manual transfer steps that increase the risk of technician error, and requires a trained technician. Amongst these, the principle shortcoming that we sought to overcome was the requirement of a trained technician. We sought to develop an automated tool that could be used in a doctor’s office by an individual with minimal training. Developing this type of tool would allow satellite doctors offices to offer IUI as a treatment option without the support of a clinic. The automated instrument may also be of interest to existing clinics based off of its speed and sperm recovery performance.

Microfluidic tools are particularly well suited for developing an automated instrument for sperm preparation. Numerous microfluidic devices that perform both motility-based and shape-based sperm selection have been developed. Microfluidic motility-based methods select sperm based on their ability to swim across streamlines^8,9^, against a flow^10^, through long channels^11^, or through filter membranes^12^. While motility-based selection is advantageous for selecting a subpopulation of only motile sperm cells with low DNA damage^11^, the selection results in the loss of a substantial portion of viable sperm cells. Since the success rate of IUI is most closely associated with the quantity of motile sperm cells^4^, motility based selection methods are not optimal for IUI^13^ since they lose so many viable sperm. Shape-based selection of sperm cells has been achieved using pinched flow fractionation^14,15^, acoustic trapping^16^, and inertial microfluidics^17^. However, all of these tools have employed flow rates and sample volumes much lower than would be required to process whole semen samples. For these reasons, there are no microfluidic-centered sperm separation techniques used clinically.

We have previously published the development of shape-based selection of sperm cells using inertial Dean focusing, although we have never demonstrated a single tool capable of processing semen samples for IUI. Past publications focused on the development of tools to select immature and immotile sperm cells from testicular biopsy samples^17–19^. Separately, we have shown the ability to select sperm cells from WBCs with a spiral channel microfluidic device^20^. However, none of these publications demonstrated a protocol able to prepare sperm for IUI; specifically, these publications did not process sperm suspended in seminal plasma, semen-native WBCs, samples with IUI-relevant concentrations of sperm, and these publications did not show IUI-relevant viability measurements of processed sperm cells. In addition, these publications have not shown a tool capable of processing a whole biological sample. Important contributions from these publications relevant to IUI sample preparation that we will build on in this work include the ability to deal with viscoelastic samples using traditional microfluidic hardware and a blueprint for the processing of other biological samples that require both a washing and separation step.

In this work, we have developed a protocol capable of preparing sperm for IUI and demonstrated an automated instrument capable of performing that protocol. Our instrument processes a diluted semen sample, and delivers a sample that is prepared for IUI with no additional post-processing required. The instrument employs repeated passes of a microfluidic chip which employs Dean forces to perform shape-based isolation of sperm cells. The instrument outperforms current clinical methods in terms of time, sperm yield, and automation. The presented applications to semen samples may be of interest to both microfluidic and clinical researchers. For microfluidic researchers, the results demonstrate a repeatable protocol for dealing with viscoelastic samples in a spiral channel at flow rates much higher than most previous studies. For clinicians, the automated instrument that may extend the accessibility of IUI by removing the clinical support required for sperm preparation.

## Materials and methods

### Dean flow theory

**Figure 1:**
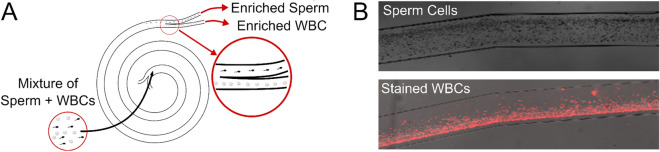
A microfluidic spiral channel can be used to separate sperm from WBCs. (**A**) Schematic representation of separation with sperm cells directed to the outer outlet of the channel and WBCs directed to the inner outlet. (**B**) Two separate photographs showing sperm cells (above) and WBCs stained with PKH26 (Sigma, MA, USA) (below) in the spiral channel show their preferential focused positions.

The chip that we employ uses a microfluidic spiral channel to create a Dean-flow based selection of sperm cells (Fig. 1). Dean focusing in Newtonian fluids has been widely explored in theory^21–24^ and application^25,26^, and essentially relies on the balancing of three inertial effects: shear gradient lift force, wall repulsive force, and Dean drag. In pressure driven flow through microfluidic channels, the shear gradient lift force drives particles away from the channel center, while the wall repulsive lift force limits the proximity of particles to a wall^27^. In curved channels, a secondary flow called Dean flow is introduced; it can be represented as counterrotating vortices in the cross sectional plane of the channel^27^. Since the magnitude of these inertial forces depends on the geometry of the target particle, a spiral channel can be used to create separation in particles based on their size^24^ or shape^28–30^. We have previously explained the mechanisms enabling the separation of sperm and WBCs^20^.

### Design and fabrication

Our chip contains a channel with a cross section of 600 µm × 100 µm (width x height). The channel forms a spiral with an inner radius of 8 mm and an outer radius of 20 mm. There are 10 total loops spaced by 1.2 mm, and the total channel length is 421 mm. The channel is operated at 1.6 mL/min. The design is consistent with guidelines proposed previously^21^ except that our aspect ratio of 6:1 is wider than these guidelines. The chips were fabricated using standard soft lithography techniques in PDMS, as we have reported previously^17^. The microfluidic chip fits on a double-wide microscope slide (25 × 50 mm). Based on the flow rate and dimensions the maximum shear rate imposed on the cells is 17,750/s and the maximum shear stress they experience is 17.76 Pa, which they experience in the 1 mm tubing.

### Clinical sample acquisition and analysis

Adult males, older than 18 years, were consented under an IRB-approved protocol at the University of Utah. Informed consent was obtained from all adult males, older than 18 years, who signed the approved, appropriate documentation. All experimental protocols were approved by the IRB at the University of Utah. For all experiments, cell concentration was counted using a Makler chamber (Irvine Scientific, CA, USA)^31,32^. Where applicable, sperm viability and motility were analyzed based on WHO 5 guidelines^32^, and DNA damage via the TUNEL assay using Apo-Direct kit (MilliporeSigma, MA, USA) with a minimum of 200 cells counted per sample. Where reported, motility reports the progressive motility. The characteristics of the eight unique patient sample processed with the automated instrument are shown in Supplementary Table S1.

### Characterizing particle focusing in the chip

We characterize the focusing of sperm and WBCs suspended at two different seminal plasma-media dilutions: > 10% v/v seminal plamsa and < 2% v/v seminal plasma. For these tests, the flow rate in the chip is controlled with two syringe pumps; one syringe pump infuses the sample while the other withdraws the sample into four equal portions. We then count the total number of each cell type in each outlet portion. Results of characterization tests compare the number of sperm and WBCs in each portion against the total number of each cell type captured. To maintain high enough cell concentrations for counting, cells are first suspended in media that is then mixed with cell-free seminal plasma to achieve the required dilution. Cell concentrations between 5 and 20 million cells/mL were used, to minimize the cell to cell interactions that could have interfered with these characterization results.

### Using the chip for automated sperm preparation

#### Instrument design and protocol

**Figure 2:**
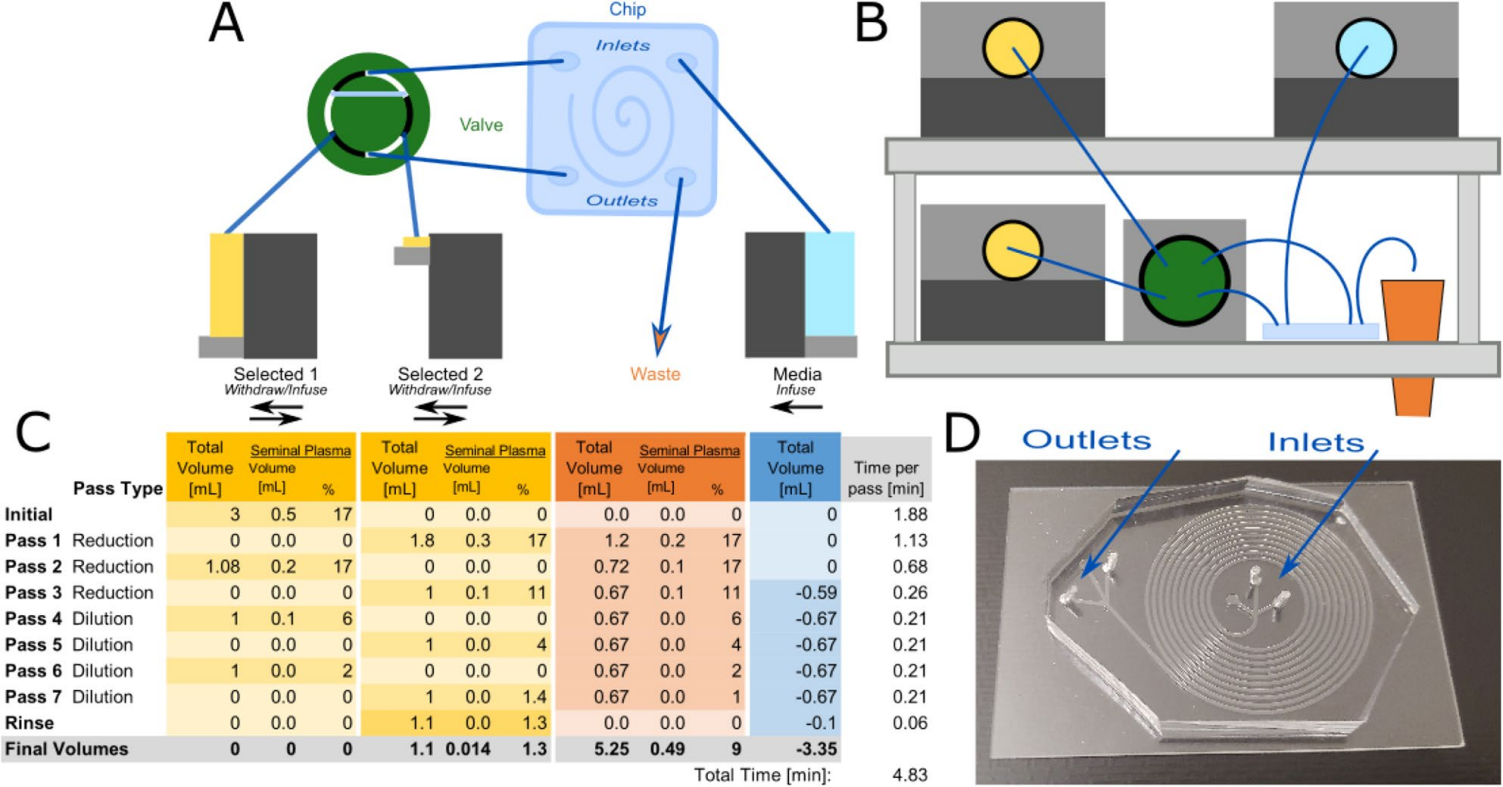
Automated Instrument and Protocol. (**A**) The components of the automated platform are shown, including three syringe pumps, one valve, one microfluidic chip, and a waste outlet. (**B**) The layout of the components in the instrument is presented. Although the valve and microfluidic chip are enlarged for clarity, the whole instrument is 30″ wide and 18″ tall. (**C**) Protocol to operate automated instrument. The initial sample contains 2.5 mL of media and 0.5 mL of seminal plasma (3 mL total). Each row represents the fluid volumes as measured after each pass of the spiral channel. Negative values in the Table represent fluid infused from the media portion into the system. (**D**) Photo of microfluidic chip.

Our automated system is shown schematically with its operating protocol in Fig. 2. The system uses Matlab to control the pumps and valve as it moves the fluid through a series of passes of the chip. The system essentially works by moving the selected portion of sample back and forth between two syringes (labeled as Selected Portion 1 and Selected Portion 2) while the waste portion is removed and media is infused from a third syringe pump. The back and forth pumping is enabled by a single chromatography valve that allows either syringe pump to direct flow to the inlet of the chip. A small tube extending from the waste portion of the chip imposes a substantial back pressure such that fluid preferentially exits the selected portion according to the flow rate of the attached syringe pump. The ratio of the infusing to withdrawing pump determines the selection percentage, which can be varied. However, for all data shown here, a selection percentage of 60% was used, based on optimization results shown in Supplementary Figure S1.

#### Instrument performance

**Figure 3:**
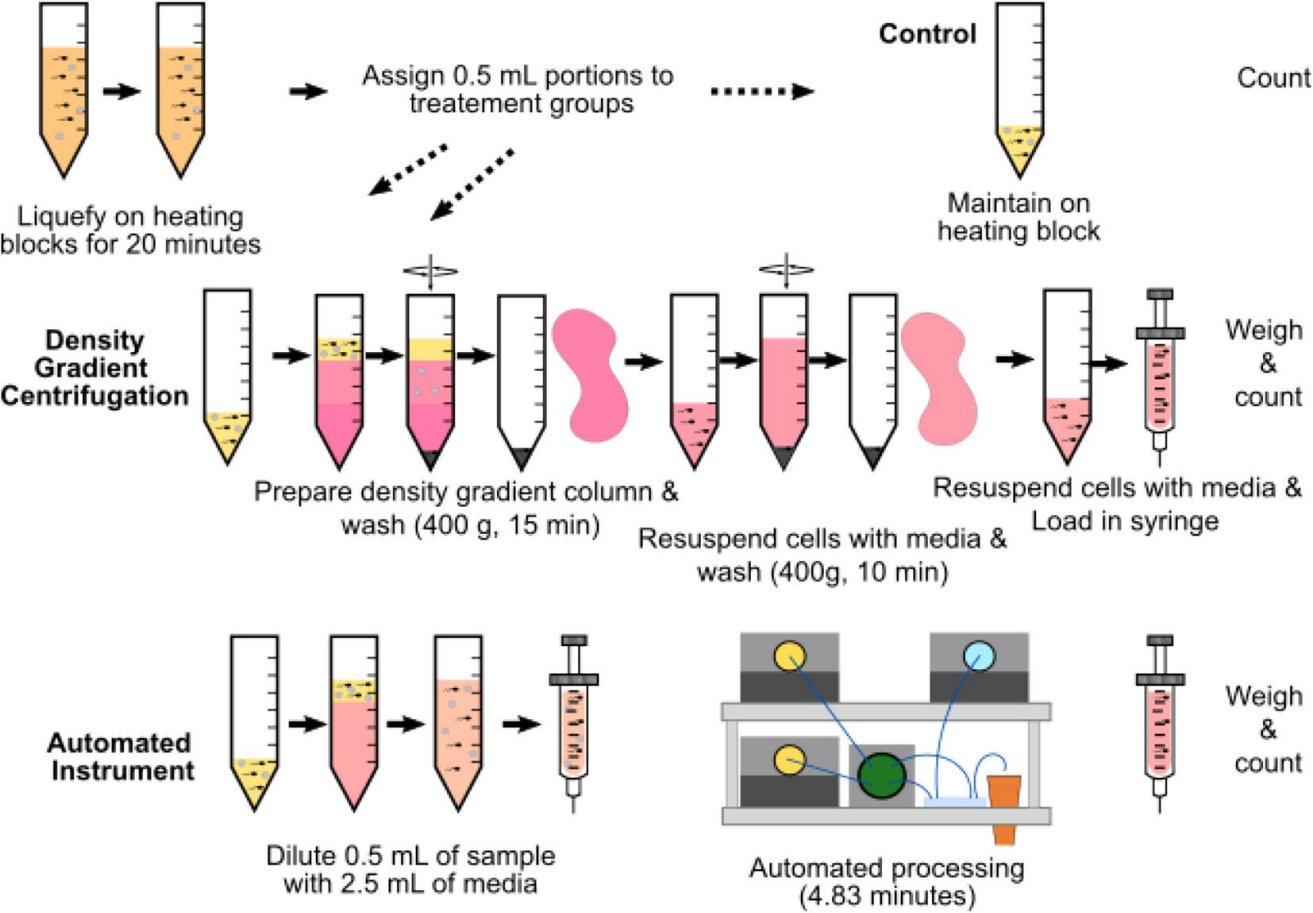
Presentation of experimental method. After the sample is liquefied, 0.5 mL portions are assigned to one of three treatment groups: The control portion remains on the heating block for the duration of the test. The density gradient centrifugation portion has two centrifugation steps before being resuspended in media. The automated instrument portion is diluted with 2.5 mL of media and then processed in the automated instrument.

In order to assess the instrument performance, we processed eight unique patient samples comparing the performance of the automated platform with a DGC (Fig. 3). Before processing, samples were allowed to liquefy by maintaining the samples at 34 °C for a minimum of 20 min. Then, the sample is split into three 0.5 mL portions: one portion is placed in an incubator with no treatment, one portion is processed with DGC, and one is processed through the microfluidic chip. DGC includes two centrifugation steps. In the first one, the sample is placed on top of 90% isolate and 35% isolate layered on top of each other (Irvine Scientific, CA, USA). After pouring off the supernatant, the sperm cells are resuspended in media to replace the isolate. After another centrifugal wash, the cells are then suspended in a small volume of media and loaded into a syringe to simulate a clinical IUI. The portion assigned to the instrument is diluted with 2.5 mL of media and loaded in a syringe which is placed in the automated instrument. For each test we report the recovery of sperm cells and the recovery of total motile sperm cells as a percentage of each type of cell in the control sample which receives no processing. The total number of cells is found by multiplying the total concentration of each cell type by the sample volume, which is determined by weight. Since the sample processed in the automated instrument is ready before the DGC processing concludes, the sample is left in the incubator at the conclusion of the test so that motility is counted simultaneously. In two cases (samples 3 and 8), a higher total sperm count was found following microfluidic processing than in the unprocessed control sample. This is the result of random sampling error driven by reduced sperm concentration in those two samples.

### Statistical analysis

All data plots show the mean value with error bars representing the standard deviation of the tests. The number of samples is contained in the figure legend. We use a paired t-test to compare the average recovery results from the DGC and automated instrument, and two-tailed *p* value is reported.

## Results and discussion

Operating the instrument for sperm preparation requires multiple passes through the chip; the first passes reduce the sample volume, the middle passes wash the sperm from the seminal plasma, and the final pass separates the sperm cells from WBCs (Fig. 2). To discuss the results, we will first discuss the characterization tests, then explain the implications of these results on the development of a protocol, and then demonstrate the ability of the instrument to perform a sperm preparation of a semen sample. We will then discuss future directions of this technology and its potential clinical impact.

### Characterizing particle focusing in the chip

**Figure 4:**
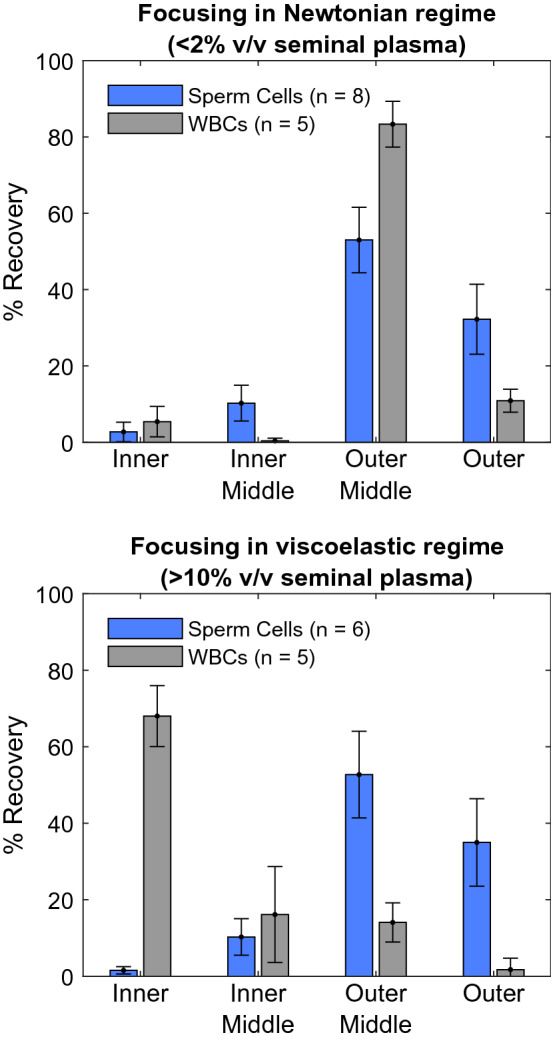
Focusing of sperm cells (blue) and WBCs (grey) in the viscoelastic and Newtonian regimes across 4 outlets. Each outlet represents 25% of the channel width. Recovery percentages are reported relative to the total number of recovered cells. All data represent the mean value of the tests, with the error bars representing the standard deviation amongst the tests. Eight samples were processed in the Newtonian regime, of which five included white blood cells. Six samples were processed in viscoelastic regime of which five had WBCs.

In the chip, we observe two unique particle focusing regimes dependent on the concentration of the seminal plasma in the suspending fluid. We characterize these regimes as occurring where seminal plasma represents < 2% and > 10% of the fluid volume (Fig. 4). Interestingly, these two regimes arise within a single chip at a single flow rate.

When the suspending fluid is < 2% v/v seminal plasma, there is a clear separation between sperm and WBCs, with the vast majority of sperm directed to the outer half of the channel and WBCs directed to the inner quarter. As we have reported previously, this separation is enabled by solely inertial effects. Sperm cells are passively aligned with the flow, primarily with their heads aligned towards the flow^33,34^ and are driven towards the horizontal center of the channel^30,35^; in that location they experience an outward-pushing Dean flow so that we observe a focused location towards the outer wall. WBCs tumble in the flow and, acting as spherical particles^28,30^, are driven towards the horizontal top and bottom of the channel^22,28^ where they experience an inward-pushing Dean flow. This enables a separation with sperm cells in the outer portion of the channel and WBCs in the inner portion of the channel.

Unfortunately, this separation is impeded at even low concentrations of seminal plasma (10% v/v). When the seminal plasma represents > 10% of the suspending fluid volume, both sperm and WBCs are directed to the outer portion of the channel, focused primarily 50–75% of the way towards the outer wall. We can describe these results by considering the viscoelastic nature of seminal plasma. The viscoelasticity of seminal plasma causes the fluid to impose an additional force on the WBCs that drives them towards the center of the channel. Although fundamentally different than the force that drives the asymmetric sperm cells to the channel center, this viscoelastic force has the same effect on the WBC focusing position as the sperm’s asymmetry; the fluid drives WBCs to the channel centerline and the Dean flow influences that focused position towards the outer wall of the channel^36,37^. Since both WBCs and sperm cells are focused primarily in the portion of the channel 50–75% of the way towards the outer wall, an efficient separation is not possible.

### Development of a protocol to process semen samples

These findings have two important implications for our automated protocol. (1) Regardless of our operating regime, we expect ~ 90% of the sperm to be directed to the outer half of the channel. This is convenient and enables a convention where the outer portion of the channel as the selected portion for all passes. (2) In our automated protocol, we will have to dilute away the seminal plasma before the separation can be achieved. To achieve dilution, before using the chip as an instrument for separation of WBCs, we use the chip as an instrument for dilution of seminal plasma. We perform the dilution by continuously selecting the sperm cells in repeated passes of the spiral channel while diluting the suspending fluid with media. Removing the seminal plasma has the effect of both enabling our microfluidic separation of WBCs and removing the seminal plasma as required for IUI.

We note that a 1 mL sample containing 2% seminal plasma will contain only 20 μl of seminal plasma, which is less than a clinical threshold (60 μl). As a result, the number of passes required is not dictated by the need to remove prostaglandins, but rather by the necessity to remove the physical fluidic influence of the seminal plasma. Additionally, the sample delivered from our instrument will likely have a lower concentration of prostaglandins than a sample prepared with a single centrifugation step.

Figure 2 includes our method to achieve the 2% seminal plasma dilution with minimal sperm loss by employing repeated passes of the chip. This protocol can be modified to bring any arbitrary concentration of seminal plasma to a concentration below 2% of the fluid volume and any arbitrarily large sample to a volume below 1 mL with a modest sacrifice in processing time required for larger samples. An example protocol to process a 2.5 and 5-mL sample with a similar starting dilution is shown in Supplementary Table S3 and Supplementary Table S3.

### Automated instrument performance

**Figure 5:**
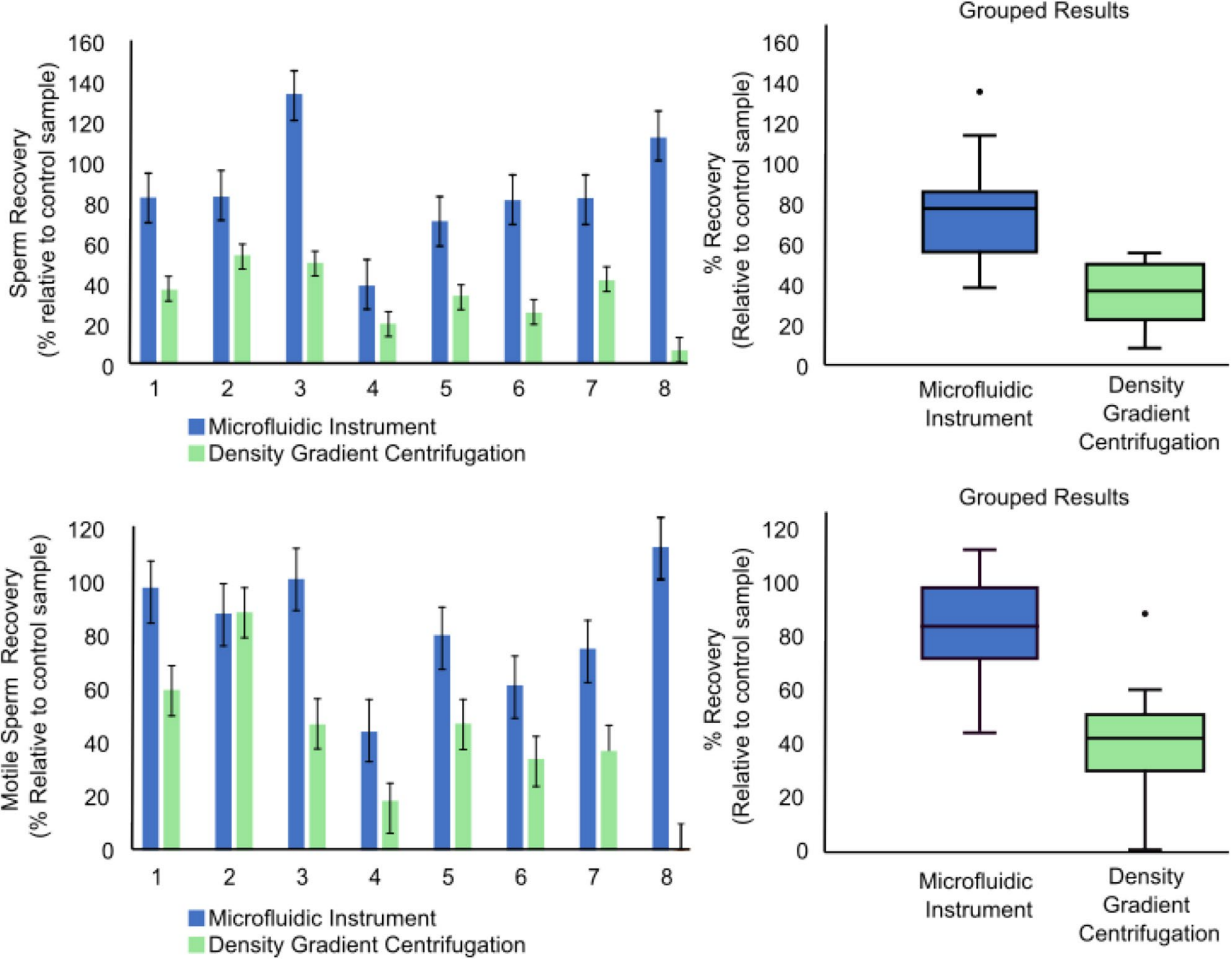
Sperm cell recovery from samples from eight males processed with the microfluidic instrument and through density gradient centrifugation. Results for all sperm cells (above) as well as motile sperm cells (below) are reported. Percentages are reported relative to the number of cells in a control sample that received no processing. Error bars, equal to 10% of the value of the highest count, represent the errors in counting. Box plots at right represent the compilation of the eight males and are offer an additional way to visualize the same data.

As presented in Fig. 2, by utilizing a combination of dilution and reduction passes, it is possible to process a 0.5 mL sample in less than 5 min. We perform this process on eight patient samples splitting each sample as described in Fig. 3. The sperm recovery from our instrument for each pass is shown in Fig. 5. We recover an average of 86% of all sperm and 82% of progressively motile sperm while the DGC recovers of 33% of all sperm and 41% of all progressively motile sperm. Since the experiments were run with split portions of the same sample, we used a paired t-test to confirm that the recovery of sperm is greater from the automated protocol for all sperm (*p* = 0.0014) and for progressively motile sperm (*p* = 0.0047).

**Figure 6:**
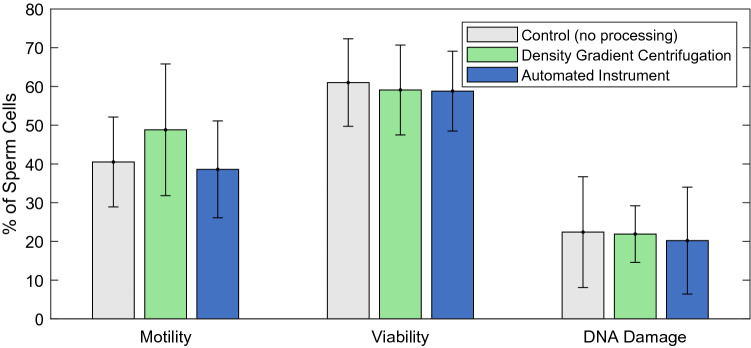
Comparison of sperm quality after processing with a density gradient (DGC) and the automated instrument. Motility (n = 10), Viability (n = 4), and DNA damage (n = 4) results are compared to a control sample that received no processing. All data represent the mean value of the tests, with the error bars representing the standard deviation amongst the tests.

To ensure that no damage was imposed on the sperm cells by our instrument, we assessed sperm viability and sperm motility based on WHO 5 guidelines^32^ and DNA damage via the TUNEL assay with a minimum of 200 cells counted per sample. From the data presented in Fig. 6, there is no apparent difference between the control or samples processed through either a DGC or our instrument. These pilot study data are not meant to provide clinical verification of the technology, but rather ensure that there is a path forward for the technology. The small number of samples limit our ability to make statistical claims about the treatment groups.

### Discussion

#### Future chip optimization

A future optimization of the chip geometry, flow parameters, or protocol could improve our performance metrics. First, other authors^17,20^ have reported tighter sperm cell focusing than has been achieved in the current work. Tighter focusing would allow for a greater removal of seminal plasma, with each pass of the chip potentially reducing the number of passes necessary, but may be limited by the high concentrations of cells present in our samples. Second, other devices have used viscoelastic focusing to manipulate cells at much higher speeds^38^. We know that our geometry continues to create flow focusing as the flow rate is increased by 10–50%, and hypothesize that a larger channel would allow flow focusing at even higher speeds. Here, our flow rate was principally limited by our fabrication techniques. Third, our instrument is only processing a very small portion of the sample at any moment in time. Operating multiple chips concurrently by placing them either in series or in parallel could substantially decrease the processing time for a sample.

Using a series of optimized chips operating in parallel on a small sample, we can envision an instrument that performs a whole sperm preparation on a liquefied sample in less than 5 min, an order of magnitude faster than currently available protocols. In that case, the rate limiting step would be liquefaction and, with a parallelized instrument, it would become even more interesting to explore methods to increase the speed of liquefaction by, for example, incorporating mechanical perturbation including sonication^39^, employing microfluidic manipulation, or exploring the extent to which we can start processing a sample before liquefaction is complete. We did not exhaustively optimize our device for this demonstration since our clinically-relevant metrics already considerably surpassed the performance of existing techniques.

#### Potential clinical utility

In our tests, our automated protocol recovers more motile sperm cells in less time than a DGC. While data collected here focused on processing a split portion of a sample, it is possible to process much larger samples with modest sacrifices in time. Protocols to process 2.5- and 5-mL samples are shown in Supplementary Tables S2 and S3. As the sample size increases, the time savings of the automated protocol decreases, although this could be rectified by parallel processing as described previously. Additionally, while our tests employed semen samples that were originally diluted to 17% of the fluid volume, it is possible to start with samples which were diluted to only 50% of the fluid volume. The fundamental limitation from our perspective was that at higher concentrations of semen, the pressure in the device increased to a point that would cause device failure, and so the dilution we employ was chosen with a factor of safety and for convenience. In the Supplementary Tables S4 and S5, we show if a smaller volume of media was used to initially dilute the sample, we could reduce the processing time from 24 to 10 min for 2.5 mL samples and from 31 to 19 min for 5 mL samples. Using different fabrication methods, larger channels, or lower flow rates could be used to help reduce the pressure associated with high seminal plasma concentrations.

Viability results presented herein show that there is no harm imposed on the sperm cells by the proposed preparation method. There are a few reasons that our processing method may improve sample characteristics across these tests relative to current clinical methods: our method takes less time, avoids centrifugal forces, and can remove more of the seminal fluid. We hope that these factors can be linked with clinically relevant sample improvements in, for example, DNA damage.

The instrument that we have described is capable of processing a semen sample in a manner that outperforms DGC across many key metrics, most notable of which is the total number of sperm cells recovered. However, one metric in which the DGC outperforms our instrument is in the percentage of selected sperm that are motile. We believe that for any given sample, our instrument captures all of the sperm that the DGC would select and additionally selects more motile sperm (while selecting additional non-motile sperm as well). Since the number of total motile sperm has been reported as the key indicator for IUI success^4^, we expect that our instrument could improve clinical outcomes, particularly in the context of samples with an especially low initial motility. However, it is conceivable that the higher concentration of non-viable sperm could interfere with pregnancy initiation. We are not aware of any data that demonstrated this, nor can we propose a specific mechanism by which that would occur, but we are interested in exploring this specific attribute either by parsing through existing data sets or by performing in vitro tests.

While the instrument described, does outperform a DGC on important performance metrics, we believe that the most apropos clinical application of this automated instrument is in smaller medical facilities, that are not currently supported by clinics with trained technicians. Here, the automated instrument, offers the ability to offer onsite IUI services without the support of a clinic and laboratory. One important shortcoming to be overcome before the technology is used in this way is that the technology should be optimized to deliver smaller sample volumes, as the optimal volume used for IUI is 0.5 mL or less.

## Conclusion

We developed an instrument that can perform an automated sperm preparation for IUI. The instrument takes as its input a diluted semen sample and produces a prepared sample ready for insemination. In our tests, the presented instrument outperforms the clinical gold standard sperm preparation across many key metrics. The preparation method recovers more sperm cells, requires less time, is tunable and automated, and avoids centrifugation, potentially improving cell viability in the final sample. The enabling technology for this preparation is repeated passes of a microfluidic chip that selects the sperm cells based on their unique shape such that they can be concentrated in a given volume of fluid or separated from round cells. The instrument presented here could have an immediate clinical impact on IUI sperm preparation as it outperforms all other methods in total motile sperm recovery. It could make IUI more accessible and potentially increase the success rate of the procedure, particularly in those whose motile sperm counts are on the low end for those recommended to attempt IUI procedures.

## Supplementary information

Supplementary information.
