# Supplementary material for: An automated instrument for intrauterine insemination sperm preparation
Source: Sci Rep. 2020 Dec 7;10:21385. doi: 10.1038/s41598-020-78390-3 (PMC7721893; doi:10.1038/s41598-020-78390-3)
Supplement: Supplementary file 1 — Supplementary information. [file 41598_2020_78390_MOESM1_ESM.docx]

**An Automated Instrument for Intrauterine Insemination Sperm Preparation**

Alex Jafek, Haidong Feng, Hayden Brady, Kevin Petersen, Marzieh Chaharlang,

Kenneth Aston, Bruce Gale, Timothy Jenkins, Raheel Samuel

**Supplementary Table S1**: Sample characteristics for eight samples processed to generate the data in Figure 5. Sample characteristics were measured on control sample which received no processing.

**Sample Characteristics**

| **Sample No.** | **Clinical Viscosity** | **Sperm Concentration**  (Million/mL) | **% Motile Sperm** |
| --- | --- | --- | --- |
| 1 | Normal | 81 | 42 |
| 2 | Normal | 159 | 39 |
| 3 | Normal | 49 | 38 |
| 4 | Normal | 40 | 15 |
| 5 | Normal | 118 | 39 |
| 6 | Normal | 81 | 52 |
| 7 | Normal | 36 | 40 |
| 8 | Normal | 41 | 4 |

**
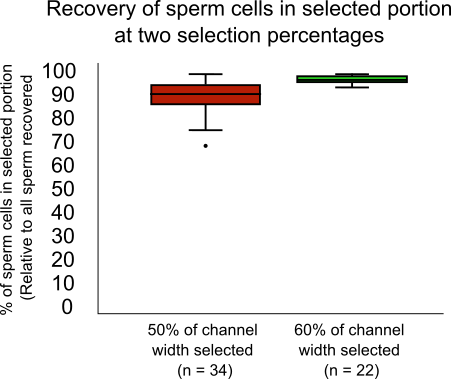
**

**Supplementary Figure S1**: Comparison of sperm recovery with selection percentage set at 50% (n = 34) and 60% (n = 22).

**Supplementary Table S2**: Protocol to process 2.5 mL sample, initially diluted with 12.5 mL of media.

**Supplementary Table S3**: Protocol to process 5 mL sample, initially diluted with 15 mL of media.

**Supplementary Table S4**: Protocol to process 2.5 mL sample, initially diluted with 3.5 mL of media.

**Supplementary Table S5**: Protocol to process 5 mL sample, initially diluted with 15 mL of media.
